# External auditory canal cholesteatoma: 20 years of experience

**DOI:** 10.1016/j.bjorl.2025.101627

**Published:** 2025-04-25

**Authors:** Federico Herranz, Julián Agustín Simkin, Federico Di Lella, Gabriela Perez Raffo

**Affiliations:** Hospital Italiano de Buenos Aires, Department of Otolaryngology, Otology Section, Buenos Aires, Argentina

**Keywords:** Cholesteatoma, Othorrea, Keratosis obturans, Otologic surgical procedures, Canaloplasty

## Abstract

**Objective(s):**

To determine the prevalence of external auditory canal cholesteatoma in the Otolaryngology service of the Hospital Italiano de Buenos Aires, and to evaluate the therapeutic approaches and their outcomes.

**Methods:**

A retrospective review was conducted of the electronic medical records of adult patients diagnosed with external auditory canal cholesteatoma between August 2004 and April 2024. Inclusion criteria were a confirmed or presumptive diagnosis of external auditory canal cholesteatoma and a minimum follow-up of six months. Variables analyzed included age, sex, disease severity (classified using the Udayabhanu HN 2018 classification), laterality, therapeutic approach, recurrence, and follow-up duration.

**Results:**

Fifty-two patients met the inclusion criteria, with an average age of 60.3 years. Most were female (57.7%). Bilateral involvement occurred in 5.8% of cases, and 86.5% of external auditory canal cholesteatoma were primary. According to the Udayabhanu HN classification, 28.8% were grade I, 63.5% were grade II, and 7.7% were grade IIIa. Conservative treatment was administered to 53.8% of patients, while 46.2% underwent surgery. The majority of surgical patients (58.3%) received canaloplasty and reconstruction, while 33.3% underwent only canaloplasty. Canaloplasty with mastoidectomy and meatoplasty was performed in 8.3% of cases. One patient experienced recurrence 13 years post-surgery.

**Conclusion(s):**

Both surgical and conservative treatments for external auditory canal cholesteatoma can lead to successful outcomes. The therapeutic approach should be tailored to the stage of the disease and patient characteristics, with early diagnosis and appropriate management essential to preventing complications.

**Level of evidence:**

4.

## Highlights

• Clinical and surgical approaches used for managing external auditory canal cholesteatoma.
• Non-surgical treatment effectively controlled 53.84% of external canal cholesteatomas.
• Advanced cases required canaloplasty, reconstruction, or mastoidectomy.
• 95.83% of surgically treated patients showed no recurrence after follow-up.
• Stage II was most common, accounting for 63.5% of cases.

## Introduction

**Fig. 1:**
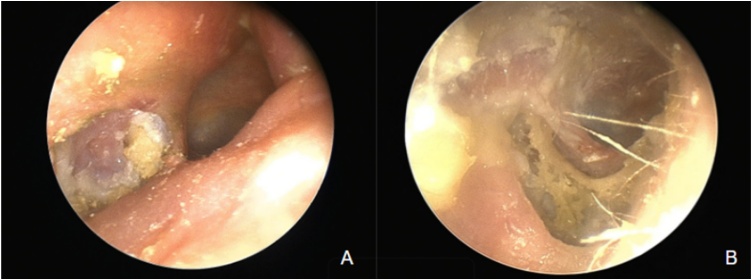
Endoscopic image of external auditory canal cholesteatoma. (A) Lesion with bone erosion on the posterior wall of the right external auditory canal. (B) Lesion with bone erosion on the floor of the right lower canal.

External Auditory Canal Cholesteatoma (EACC) is characterized by the invasion of squamous tissue into a localized area of bone erosion within the canal. Clinically, it often presents with otorrhea and chronic, dull pain. It was first described in 1850 by Toynbee, but since then it has been confused with keratosis obturans. It commonly occurs in patients between the ages of 40 and 75 and is very rare in younger patients. Keratosis obturans results from the accumulation of large plugs of keratin desquamation without bone invasion.^1^ The presumptive diagnosis is made based on physical examination, where otomicroscopy or otoendoscopy reveals epidermal tissue in the EAC, generally associated with exposed bone (Fig. 1).

EACC can be classified as either primary (idiopathic) or secondary. Primary EACC occurs without an identifiable cause, whereas secondary EACC is associated with risk factors such as previous ear surgery, radiotherapy, chemotherapy, or external auditory canal stenosis.^2^, ^3^

Both expectant management and surgical options are valid for EACC. In general, it is accepted that patients can be managed in the office if the full extent of bone erosion is visible and the patient is not in pain.^4^ However, some authors argue that bone erosion may extend beyond what is visible in the canal, thus always recommending surgery.^5^ High-Resolution Computed Tomography (HRCT) is essential for diagnosing and staging EACC, enabling detailed visualization of bony erosion and disease extent.^6^ EACC classification can be clinical-radiological or histological. Naim et al.^7^ proposed a histological classification with four stages: I) Focal epithelial hyperplasia, II) Periostitis, III) Bone erosion, and IV) Erosion of adjacent structures. However, it lacked clinical correlation and surgical guidelines.

Shin (2010) developed a radiological classification that required no pathological analysis, making it a pre-surgical option. The pathology was divided into four stages: 1) Limited to the external auditory canal; 2) Involving the tympanic membrane/middle ear; 3) Creating a defect in the canal with mastoid air cell involvement; and 4) Extending beyond the mastoid bone.^8^

In 2018, HN et al.^9^ introduced a clinical classification with stage-specific management: 1) EACC without bone erosion/middle ear extension; 2) EACC with bone erosion, with or without middle ear extension; and 3) EACC with bone erosion + adjacent structure extension (temporomandibular joint, mastoid, facial canal, jugular bulb, dura mater). Stage 3I is subdivided into a) Without complications and b) With complications (e.g., facial paralysis). Based on this latest classification, the objective of this study was to determine the prevalence of EACC in the Hospital Italiano de Buenos Aires and to describe the therapeutic approaches taken, evaluating their outcomes.

## Methods

A retrospective study was conducted by reviewing the electronic medical records of all patients diagnosed with EACC between August 2004 and April 2024. EACC was defined as the presence of cholesteatoma originating in the external auditory canal, without evidence of having originated elsewhere, such as the middle ear or mastoid. A help desk was created to generate a list of patients with “External auditory canal cholesteatoma” as a diagnosis within the electronic medical record.

### Inclusion criterio

Confirmed or presumptive (in patients who did not undergo surgery) diagnosis of EACC. Follow-up of more than 6 months after the therapeutic approach was adopted.

### Exclusion criterio

Missing data.

Lack of follow-up.

The variables evaluated were the age of presentation, medical history, gender, the degree of cholesteatoma according to Udayabhanu HN’s 2018 classification,^5^ laterality, location, therapy, recurrence, and follow-up time. All patients underwent audiological evaluation as part of routine clinical assessment. However, audiometric data were not included in the analysis, as hearing outcomes were not a primary objective of this study. Primary EACC was defined as cases occurring in patients without prior otologic surgery, chemotherapy, radiotherapy, or external auditory canal stenosis.

All surgical procedures were performed by two experienced otologic surgeons, Dr. Di Lella, Federico and Dr. Perez Raffo, Gabriela, both of whom have extensive expertise in the management of otologic conditions.

Our study was approved by the Ethics Committee for Research Protocols (CEPI) of Hospital Italiano de Buenos Aires, protocol number #11879 in the PRIISA system of the Government of the City of Buenos Aires.

## Results

Through the help desk at Hospital Italiano de Buenos Aires, 67 patients with a diagnosis of “External auditory canal cholesteatoma” were identified. After exclusions, 52 patients were included in our study, with a total of 55 affected ears. The prevalence rate was 2 per 1000 otological patients (52 patients with EACC out of 23,216 otological consultations during the study period).

Demographic data are detailed in Table 1. Our group showed a female predominance, with a total of 30 women (57.7%) and 22 men (42.3%). The mean age was 60.3 years, with a range from 12 to 98 years. Regarding laterality, only 3 patients (5.8%) had bilateral pathology, leading to a total of 55 affected ears. The rest were divided between the right ear (28 patients, 53.8%) and the left ear (21 patients, 40.4%).

**Table 1:** Patients included in the study.

	*N*	Percentage (%)
Total patients	52	
Total affected ears	55	100
Females	30	57.7
Age		
Average	60.3	
Range	12‒98	
Laterality		
Right	28	53.8
Left	21	40.4
Bilateral	3	5.8
Grade		
I	15	27.2
II	36	65.5
IIIa	4	7.3
IIIb	0	0
Management		
Clinical follow-up	31	56.4 (of affected ears)
Surgical	24	43.6 (of affected ears)
Canaloplasty	8	33.3 (of surgical ones)
Canaloplasty + reconstruction	14	58.3 (of surgical ones)
Canaloplasty + reconstruction + mastoidectomy + meatoplasty	2	8.3 (of surgical ones)

Forty-five patients (86.5%) had primary or spontaneous EACC. The remaining 13.5% (7 patients) were presumed to be secondary due to their medical history: 3 patients had records of prior tympanoplasties, 2 patients had undergone chemoradiotherapy for medulloblastoma, and 2 patients had external auditory canal stenosis.

All patients were classified according to Udayabhanu HN’s 2018 clinical-radiological classification. Some patients in our group did not have imaging in their electronic medical records, so they were assumed to have no bone erosion. Fifteen patients had Grade I (27.2%); 36 had Grade II (65.5%); and 4 patients were Grade IIIa (7.3%). No Grade IIIb patients were found.

Regarding location, 30 patients (57.7%) had involvement of the floor of the EAC, and 15 (28.8%) had involvement of the posterior wall. Four patients (7.7%) had anterior wall involvement, and only one (1.9%) had involvement of the roof. Two patients (3.8%) had involvement of more than one wall.

Twenty-eight patients were managed in the office without surgical intervention, with control of the disease. Follow-up visits were conducted semi-annually with variable adherence, during which EAC cleanings were performed. The reasons for this non-surgical management varied: in some patients, it was due to surgical risk related to comorbidities and age; in others, it was the patient’s decision; and finally, in some, it was due to the ability to fully control the disease in the office.

The remaining 24 patients underwent surgery. In 21 patients, a retroauricular approach was used, and in the remaining 3, an endaural approach. In 14 of them (58.3%), canaloplasty + reconstruction was performed. Various types of grafts were used for the reconstruction, including perichondrium, temporal muscle fascia, and skin. In 8 patients (33.3%), only canaloplasty was performed without the need for reconstruction. In two cases (8.3%), canaloplasty + reconstruction was associated with mastoidectomy and meatoplasty.

The median follow-up time was 4.5 years, with a range from 6 months to 21 years. Only one patient experienced a recurrence of the disease (1.92%). This patient had undergone canaloplasty + reconstruction and had a recurrence 13 years postoperatively. The recurrence was managed with successive cleanings in the office. Three of the 24 surgically treated patients (12.5%) developed tympanic membrane perforations as a postoperative complication. The perforations were detected at 4 months, 3 years, and 7 years post-surgery in each respective patient. In two cases, the initial treatment for EACC was canaloplasty. Both patients had a history of chemotherapy and radiotherapy. Tympanoplasty using tragal perichondrium was performed three years after the initial surgery in both cases, with successful outcomes.

The third patient had primary EACC with lesion extension near the annulus. This patient was initially treated with canaloplasty but developed an inferior tympanic membrane perforation over time. Surgical repair with tympanoplasty was performed seven years after the initial procedure, following the same technique used in the previous two cases. Postoperative evolution was favorable in all three patients.

## Discussion

Cholesteatoma is a keratinizing squamous cystic structure associated with bone erosion, typically found in the middle ear but sometimes in the external auditory canal. The estimated incidence of External Auditory Canal Cholesteatoma (EACC) is 0.1%‒0.5% of otological patients.^9^ Our rate, 2 per 1000, aligns with this estimate.

Though rare, recognizing EACC is crucial, as its management differs from neoplasms, keratosis obturans, or malignant otitis externa. It is classified as primary or secondary, with secondary cases arising from stenosis, exostosis, trauma, surgery, tumors, or radiotherapy. In primary cases, factors like microtrauma, compacted cerumen, focal osteitis, hypoxia-induced angiogenesis, and erratic keratin deposits have been proposed.^10^, ^11^ Chronic cotton swab use is considered a risk factor, though it is unclear whether it causes EACC or if otorrhea is the trigger.

In the majority of studies, a distinction is made between primary and secondary cholesteatomas: Udayabhanu HN and colleagues^8^ presented 51.6% of primary and 48.3% of secondary cases (with postoperative cases being the most common among the latter). These authors^8^, ^12^ described a cohort of 13 patients with 61.53% having spontaneous EACC and 38.47% being secondary. In our study, the percentage of spontaneous cases was much higher (86.5%). This may be explained by the fact that the two previously mentioned studies, like most, only included patients who underwent surgical treatment, while our study also included patients managed in an outpatient setting. If we only consider the surgical patients, the percentage of secondary EACC rises from 13.5% to 27.2%, though this is still lower than in other studies.^8^, ^12^ This may also be explained by a potential bias due to missing data in the medical records, such as a history of temporal trauma, a common cause in the cited publications.

Heilbrun, in his clinical and imaging study of the disease, presented 13 cases with 100% showing bone erosion.^12^ Udayabhanu HN and colleagues found 64.5% of patients with erosion during intraoperative review.^8^, ^12^ Our group reported 71.2% of cases with bone erosion. The fact that our percentage and Udayabhanu HN's are lower than Heilbrun's may be due to earlier detection of the disease, allowing for identification before advanced stages.

Shin reported 29 patients who underwent surgery, with 48% classified as grade I, 10% as grade II, 34% as grade III, and 7% as grade IV according to his classification system. His work is one of the few that presents a predominance of early stages since these are generally asymptomatic and often go unnoticed.^7^

Udayabhanu HN^8^ presented a new classification based on radiological evaluation and surgical confirmation, as they argue that the latter is more reliable for determining the involvement of adjacent structures. This is the classification we used with some distinctions: in a large portion of our group, a non-surgical approach was adopted, meaning there was no surgical confirmation of the grade; many patients did not undergo CT scans, so they were staged clinically. Our group had 27.2% in grade I (35.4% in the original study), 65.5% in grade II (25.8% in the original study), and 7.3% in grade III (38.7% in the original study). If we only consider surgical patients, the numbers change to 8.3%, 79.16%, and 12.5%, respectively. Although grade III increases when we include surgical patients, the percentage remains lower than in the original study. This could be due to more aggressive and earlier surgical indications, allowing the disease to be detected at less advanced stages.

The most affected area in our series was the floor of the External Auditory Canal (EAC) in 57.7%, followed by the posterior wall in 28.8%. In Udayabhanu HN's series of 31 patients, 16 (51.6%) were located on the floor of the EAC, 7 (22.5%) on the posterior wall, and 4 (12.9%) on the anterior wall. Only two patients (6.4%) had involvement of the superior wall and attic.^8^

Classically, both conservative medical and surgical treatments have been valid options for managing EACC.^1^, ^8^, ^12^ Early stages can be managed through periodic cleanings in the outpatient clinic, with or without local anesthesia. This requires patient cooperation and logistical availability for follow-up appointments. Patients with significant comorbidities who may not tolerate surgical intervention and those who prefer non-surgical management while maintaining good adherence to follow-up care may also benefit from a conservative approach.^13^ Surgical treatment is recommended for cases with progressive disease or extensive bone erosion, particularly when there is middle ear involvement.^6^ Additionally, surgery is indicated for patients with persistent symptoms such as recurrent otorrhea, pain, or hearing loss despite conservative treatment.^14^ Situations where the disease poses a risk of complications, such as facial nerve involvement or erosion of adjacent structures, also warrant surgical intervention.^15^ The previously mentioned case series only included surgical patients. In our report, 28 patients (56.4%) were managed clinically. None of them experienced complications or required a change in management (from clinical to surgical).

The remaining 24 patients (43.6%) were surgically managed. Various surgical techniques have been described (transcanal, endaural, retroauricular). The procedure should aim to excise the lesion for deferred pathological analysis and reconstruct the EAC to create a self-cleaning canal (canaloplasty, closed mastoidectomy), or in more advanced cases, an open mastoidectomy may be necessary. In our series, most cases were approached retroauricularly, with only three endaural approaches for grade II lesions (bone erosion without middle ear involvement) of limited extension.

**Fig. 2:**
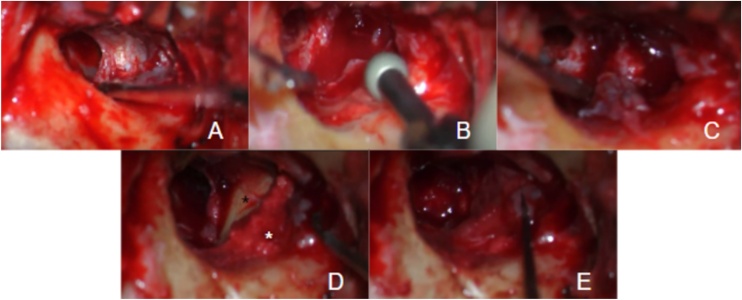
Retroauricular approach to the right ear. (A) Cholesteatomatous tissue eroding the floor of the external auditory canal is evident. The normal tympanic membrane is also visualized in the background. (B) Canaloplasty is performed to expose the entire pathology. (C) Resection of the lesion is conducted. (D) Reconstruction is performed using tragal cartilage (black asterisk) and bone paste (white asterisk). (E) A perichondrial graft is placed over the previous materials.

**Fig. 3:**
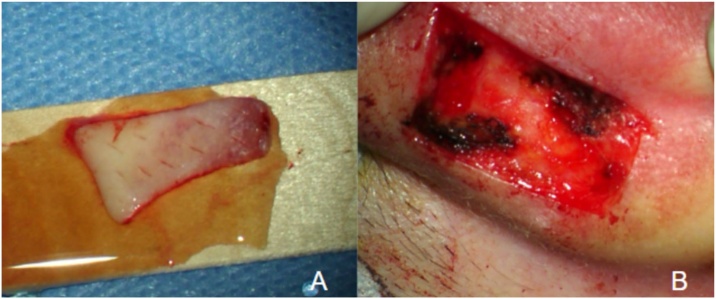
(A) Free skin graft. (B) Donor site from the auricular pavilion.

In 14 patients (58.3%), canaloplasty + reconstruction was performed. Different types of grafts were used for reconstruction, including perichondrium, temporalis muscle fascia, and skin. Konishi and colleagues recommend multilayer reconstruction, especially in advanced stages. They used bone paste, postauricular muscle-periosteal flaps, autologous cartilage, and temporalis muscle fascia.^3^ Our reconstructions varied, with some patients receiving a single-layer graft and others with more extensive disease receiving multiple layers. The only canaloplasty with endaural reconstruction used tragal cartilage and perichondrium, a valid option that requires a small incision that is generally not visible. These materials were also used in retroauricular approaches, as shown in Fig. 2. Temporalis muscle fascia was obtained through the same retroauricular approach and used as a graft in five patients. In four patients, free skin grafts were used, a technique that has been described in the literature.^4^ Our technique involved harvesting a skin patch from the posterior aspect of the auricle after local infiltration, creating fenestrations, and then placing it over the EAC defect (Fig. 3). In eight patients (33.3%), only canaloplasty was performed without the need for reconstruction. The goal was to drill the walls of the EAC until reaching healthy bone. This was done through both endaural and retroauricular approaches. The presence of the third portion of the facial nerve, which runs lateral to the annulus plane in 70% of cases and is always located in the posteroinferior quadrant, should be considered as it poses the highest risk of injury.

In two cases (8.3%), canaloplasty + reconstruction was associated with mastoidectomy and meatoplasty. Both cases were classified as grade IIIa: one had invasion of mastoid air cells, and the other had involvement of both mastoid air cells and the facial nerve at the stylomastoid foramen.

**Fig. 4:**
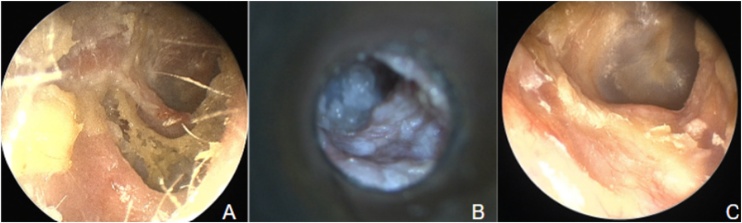
(A) Preoperative endoscopy. (B) Postoperative otomicroscopy (2-months). (C) Postoperative endoscopy (4-months).

Of the 24 surgically managed patients, only 1 (4.17%) experienced recurrence of the lesion. In 23 patients (95.83%), disease control was achieved with no evidence of recurrence at follow-up. Our success rates are comparable to those observed in other series: Udayabhanu HN reported no recurrence in any of his 31 patients.^8^ Konishi reported only one recurrence (3.57%) in his series of 28 patients.^3^ Of the 29 patients reported by Shin, 2 (8.33%) experienced recurrence. Both had low-grade EACC, and it was suggested that hidden disease might have been missed during the first surgery.^2^ Our only patient who had a recurrence did so 13 years after the surgical procedure and was managed with office cleanings. This patient also developed EACC in the contralateral ear, which was managed conservatively at the patient's request, as she did not want to undergo another surgical procedure. Fig. 4 illustrates the postoperative evolution of one of our patients compared to the preoperative lesion.

The overall complication rate was 12.5%, with tympanic membrane perforation as the only postoperative issue. Two of the three affected patients had prior chemotherapy and radiotherapy, likely contributing to impaired healing and delayed epithelialization. This supports previous findings that oncologic treatment may increase postoperative complications due to vascular compromise and tissue fragility.^15^, ^16^

The third patient had primary EACC near the annulus, which may have influenced the development of an inferior tympanic membrane perforation. This highlights the importance of anatomical factors in surgical planning, as areas with limited vascular supply may be more prone to complications.

Udayabhanu HN reported three tympanic perforations (9.67%),^8^ and Konishi reported 4 (14.28%).^3^ Our three patients underwent tympanoplasty with favorable outcomes, reinforcing the safety and efficacy of canaloplasty for EACC treatment.

This study provides novel insights into the management of EACC by including both surgical and non-surgical patients, which is rarely addressed in previous literature. Most published series focus exclusively on surgically treated cases, whereas our study incorporates a significant proportion of patients who were managed conservatively. This allows for a broader understanding of the natural progression of EACC and the feasibility of long-term disease control without surgery in selected cases.

Another key contribution of our study is the low recurrence rate observed after surgical treatment (4.17%), which is consistent with previous reports but reinforces the effectiveness of different surgical approaches, including canaloplasty with or without reconstruction. Additionally, we report a long-term follow-up period, which provides valuable information on the durability of treatment outcomes.

## Conclusion

EACC is a condition with a prevalence of 1–5 cases per 1000otological consultations, with an unclear etiology. The management approach can be either surgical or non-surgical, with early stages often treated conservatively. Surgical options depend on the extent of bone erosion and the involvement of adjacent structures, with a high rate of disease control following postoperative interventions.

## Funding

This research has not received specific funding from public sector agencies, commercial sectors, or non-profit organizations.

## Declaration of competing interest

The authors declare no conflicts of interest.
